# Unpacking the Obesity Paradox in Lung Cancer: A Nationwide Analysis of In-Hospital Mortality From the US National Inpatient Sample (2020-2022)

**DOI:** 10.7759/cureus.115771

**Published:** 2026-09-04

**Authors:** Rohith Kode, Anirudh Srinivas Teja Peela, Karishma Reddy Jalapu, Bhavya Bommadi, Ajay Goud Nattala, Jai Hind Reddy Nalla, Suhrut Nag Pentakota, Sai Kiran Kuchana, Rohit Ganduboina, Sridhar Reddy Patlolla

**Affiliations:** 1 Internal Medicine, Kakatiya Medical College, Warangal, IND; 2 Internal Medicine, NRI Institute of Medical Sciences, Visakhapatnam, IND; 3 Internal Medicine, St. Francis Medical Center, Lynwood, USA; 4 Internal Medicine, Centinela Hospital Medical Center, Inglewood, USA; 5 Internal Medicine, Carle Foundation Hospital, Urbana, USA; 6 Internal Medicine, Shasta Regional Medical Center, Redding, USA; 7 Internal Medicine, King George Hospital, Visakhapatnam, IND; 8 Neurology, Abbott Northwestern Hospital, Allina Health, Minneapolis, USA; 9 Cardiac Surgery, Cardiovascular Institute, Northwell Health, New York City, USA; 10 Internal Medicine, Mission Hospital, Asheville, USA

**Keywords:** administrative data, cachexia, in-hospital mortality, lung cancer, national inpatient sample, obesity paradox

## Abstract

Background

Lung cancer remains the leading cause of cancer-related mortality worldwide. Although obesity is an established risk factor for multiple malignancies, numerous studies have reported an "obesity paradox," wherein patients with obesity demonstrate improved survival compared with normal-weight or underweight individuals. Whether this survival advantage reflects a true protective biological mechanism or methodological artifacts such as reverse causation driven by cancer cachexia and selection bias remains controversial, particularly within the acute inpatient setting. We evaluated the independent association between documented obesity and in-hospital mortality among lung cancer hospitalizations.

Methods

We performed a retrospective cross-sectional analysis of the US National Inpatient Sample (2020-2022). Adult hospitalizations (age ≥18 years) with a diagnosis of lung cancer were identified and dichotomized by the presence or absence of an International Classification of Diseases, 10th Revision, Clinical Modification (ICD-10-CM) obesity code; the unit of analysis was the hospitalization rather than the individual patient, and the exposure was documented obesity coding rather than measured adiposity. Baseline demographics, comorbidities, and in-hospital complications were compared using the chi-squared test (categorical variables) and the Mann-Whitney U test (continuous variables). The primary outcome was in-hospital mortality; multivariable logistic regression adjusted for demographic, clinical, and hospital-level covariates.

Results

Of 61,963 unweighted lung cancer hospitalizations, 7,065 (11.4%) carried an obesity code. Obese patients were younger (median: 68 vs. 70 years) and more often female (4,090/7,065 (57.9%) vs. 28,011/54,898 (51%)). They had a higher prevalence of hypertension, diabetes, dyslipidemia, and obstructive sleep apnea (1,703 (24.1%) vs. 2,910 (5.3%)) but a lower weighted Elixhauser score (median: 10 vs. 16). In-hospital mortality was lower in the obese group (278 (3.9%) vs. 3,746 (6.8%); p<0.001). After adjustment, an obesity code was independently associated with 33% lower odds of in-hospital death (adjusted odds ratio (aOR): 0.67; 95% CI: 0.58-0.77; p<0.001).

Conclusions

Documented obesity coding was independently associated with lower in-hospital mortality among lung cancer hospitalizations. However, the low sensitivity of administrative obesity coding, a binary exposure that cannot separate normal-weight from cachectic patients, and the susceptibility of hospitalized cohorts to collider and reverse-causation bias mean this association should not be interpreted as a protective effect of adiposity. The paradox is most parsimoniously explained by confounding by illness severity rather than a therapeutic target, and the finding should be interpreted as an association involving documented obesity coding rather than an effect of adiposity itself.

## Introduction

Lung cancer remains the most frequently diagnosed malignancy and the single largest contributor to cancer mortality worldwide, accounting for roughly 2.5 million incident cases and 1.8 million deaths in 2022 alone [1,2]. Despite substantial advancements in low-dose computed tomography (CT) screening, targeted therapies, and immune checkpoint inhibitors, the overall five-year survival remains below 25% across most healthcare systems, and the disease continues to drive a substantial inpatient healthcare burden. Identifying host factors and metabolic determinants that influence clinical outcomes is therefore critical for improving risk stratification and patient management.

Excess adiposity is one such factor, but its role in lung cancer is genuinely contested. At the population level, higher body mass index (BMI) is causally associated with the incidence of at least 13 cancers, and the International Agency for Research on Cancer has formally recognized body fatness as carcinogenic [3,4]. Lung cancer, however, is a conspicuous exception: epidemiological studies consistently report an inverse association between BMI and both lung cancer incidence and mortality [5,6]. This so-called "lung cancer paradox" has been observed across surgical cohorts, where higher preoperative BMI predicts better long-term survival after the resection of non-small cell lung cancer [7-9]; across immunotherapy cohorts, where obesity is independently associated with longer overall survival on checkpoint inhibitors [10]; and in large meta-analyses spanning millions of oncology patients [11,12].

The interpretation of this paradox nevertheless remains contested. BMI is a crude proxy for adiposity, the relationship between BMI category and all-cause mortality is non-linear in the general population [13,14], and Mendelian randomization analyses suggest that much of the apparent protective signal attenuates once the causal architecture linking smoking, weight, and cancer is modelled [15]. Studies restricted to diseased or hospitalized populations are also structurally vulnerable to collider-stratification and selection bias [16-19]. These methodological considerations are examined in detail in the Discussion.

Most existing evidence derives from surgical or ambulatory survival cohorts. Far less is known about how obesity relates to acute, in-hospital mortality among unselected lung cancer admissions, precisely the setting in which frailty, cachexia, and acute physiological decompensation are most visible. The objective of this study was to evaluate the independent association between documented obesity and in-hospital mortality among lung cancer hospitalizations in the National Inpatient Sample from 2020 to 2022. We hypothesized that a documented obesity diagnosis would be independently associated with lower odds of in-hospital mortality. Throughout, the exposure is defined as documented obesity coding rather than directly measured adiposity or true obesity status, and the unit of analysis is the hospitalization rather than the individual patient.

This work was previously presented as a meeting abstract at the American Association of Physicians of Indian Origin (AAPI) Annual Convention.

## Materials and methods

Data source

We analyzed the National Inpatient Sample for the years 2020-2022. The National Inpatient Sample, sponsored by the Agency for Healthcare Research and Quality within the Healthcare Cost and Utilization Project (HCUP), is the largest publicly available all-payer inpatient database in the United States, approximating a 20% stratified sample of discharges from community hospitals, from which weighted national estimates of more than 35 million hospitalizations annually can be derived [20,21]. Each record represents a single hospitalization rather than a unique patient, and all identifiers are de-identified; the analysis was therefore exempt from institutional review board approval.

Inclusion and exclusion criteria

Hospitalizations were eligible for inclusion when they occurred during the calendar years 2020 through 2022, involved an adult patient aged 18 years or older at the time of admission, and carried a diagnosis of primary malignant neoplasm of the bronchus or lung, identified by the relevant International Classification of Diseases, 10th Revision, Clinical Modification (ICD-10-CM) codes (C34.00-C34.92) in any listed diagnosis field. Within this eligible cohort, a hospitalization was classified as obese when any listed diagnosis field contained an ICD-10-CM code for obesity or a documented BMI in the obese range (E66.0-E66.9 and/or Z68.30-Z68.45) and as non-obese otherwise. This binary definition mirrors common practice in National Inpatient Sample-based research but represents a substantial limitation, because it collapses normal-weight, overweight, and underweight patients into a single reference group and depends entirely on the fidelity of hospital coding.

Hospitalizations were excluded when age was missing, when in-hospital mortality as captured by discharge disposition was not recorded, or when data required for the primary analysis, such as sex, were missing. Any record that met one or more of these conditions was removed before analysis, and the remaining eligible hospitalizations constituted the final analytic cohort.

Covariates and outcomes

Baseline covariates included age, sex, race/ethnicity, primary payer, median household income by ZIP-code quartile, admission year, admission type (elective vs. non-elective), and hospital characteristics (bed size, region, and location/teaching status). Comorbidities were captured using ICD-10-CM diagnosis codes, and overall burden was summarized with the Elixhauser comorbidity system and its van Walraven weighted point score [22,23]. The primary outcome was in-hospital mortality. Secondary descriptors included length of stay, total hospital charges, and discharge disposition.

Statistical analysis

Continuous variables, which were non-normally distributed, are presented as medians with interquartile ranges (IQR) and compared using the Mann-Whitney U test; categorical variables are presented as counts with percentages (n (%)) and compared using the Pearson chi-squared (χ²) test. For each comparison, the test statistic (χ² or Mann-Whitney U) is reported alongside the p-value. A multivariable logistic regression model was constructed to estimate the independent association between documented obesity and in-hospital mortality, adjusting for demographic, socioeconomic, hospital-level, and clinical covariates; results are reported as adjusted odds ratios (aOR) with 95% confidence intervals (CI) and the corresponding Wald test statistic. A two-sided p<0.05 was considered statistically significant. Given the very large sample size, statistical significance was interpreted in the context of effect size rather than p-values alone.

Analyses were conducted on unweighted discharge records. The National Inpatient Sample complex survey design, that is, discharge weights (DISCWT), sampling strata (NIS_STRATUM), and hospital clustering (HOSP_NIS), was not incorporated, and no survey-specific estimation procedures were applied. All counts, percentages, and model estimates reported here therefore describe the unweighted analytic sample of sampled discharges and should not be interpreted as weighted national estimates or as nationally representative population figures. The implications of this analytic choice for variance estimation and generalizability are addressed in the limitations.

Covariates entered into the multivariable model comprised two conceptually distinct groups. Baseline characteristics and pre-existing comorbidities, namely, age, sex, race/ethnicity, primary payer, income quartile, admission year, admission type, hospital characteristics, hypertension, diabetes, dyslipidemia, obstructive sleep apnea, congestive heart failure, cardiac arrhythmia, atrial fibrillation, coronary artery disease, prior percutaneous coronary intervention, pulmonary hypertension, tobacco use, deficiency anemia, alcohol abuse, renal failure, and hepatic failure, were present on or before admission. A second group of variables, such as major adverse cardiac events, stroke, acute myocardial infarction, major bleeding, fluid and electrolyte disorders, and respiratory failure, cannot be reliably distinguished in the National Inpatient Sample as pre-existing conditions versus events arising during the index hospitalization, because the database does not consistently capture present-on-admission status for these diagnoses. Estimates for this second group are reported for transparency but are susceptible to overadjustment, since conditioning on events that occur after admission and lie on the causal pathway to death can bias the estimated association; they are therefore not interpreted as baseline predictors of mortality.

## Results

Baseline characteristics

Among 61,963 unweighted lung cancer hospitalizations, 7,065 (11.4%) carried an obesity code, and 54,898 (88.6%) did not. Obese patients were younger (median age: 68 (IQR: 62-74) vs. 70 (IQR: 63-77) years; p<0.001) and more likely to be female (4,090/7,065 (57.9%) vs. 28,011/54,898 (51%); p<0.001). Racial composition differed significantly, with fewer Asian (71 (1%) vs. 2,470 (4.5%)) and more White (5,581 (79%) vs. 41,228 (75.1%)) patients in the obese group (p<0.001). Despite an identical median length of stay of four days, obese patients incurred roughly 18% higher median charges ($85,318 (IQR: $53,840-$138,278) vs. $72,428 (IQR: $41,994-$120,942); p<0.001). Critically, in-hospital mortality was markedly lower in the obese group (278 (3.9%) vs. 3,746 (6.8%); p<0.001). Differences were also seen across income quartiles, payer mix, and hospital characteristics (Table 1).

**Table 1:** Baseline demographic and hospital-level characteristics Data are presented as n (%) for categorical variables and median (IQR) for continuous variables. Test = statistical test applied: χ²: Pearson chi-squared; MWU: Mann-Whitney U; IQR: interquartile range

Characteristic	Non-obese (N=54,898)	Obese (N=7,065)	Test statistic	P-value	Test
Age, y, median (IQR)	70 (63-77)	68 (62-74)	-11.7	<0.001	MWU
Sex, n (%)					
Male	26,887 (49)	2,975 (42.1)	118.2	<0.001	χ²
Female	28,011 (51)	4,090 (57.9)	118.2	<0.001	χ²
Race, n (%)					
White	41,228 (75.1)	5,581 (79)	51.4	<0.001	χ²
Black	6,752 (12.3)	869 (12.3)	0.0	0.95	χ²
Hispanic	2,855 (5.2)	403 (5.7)	3.2	0.07	χ²
Asian	2,470 (4.5)	71 (1)	194.3	<0.001	χ²
Other/unknown	1,593 (2.9)	141 (2)	18.9	<0.001	χ²
Payer, n (%)					
Medicare	35,354 (64.4)	4,564 (64.6)	0.1	0.75	χ²
Medicaid	5,106 (9.3)	551 (7.8)	17.0	<0.001	χ²
Private	11,583 (21.1)	1,618 (22.9)	12.1	<0.001	χ²
Other/self-pay	2,855 (5.2)	332 (4.7)	3.2	0.074	χ²
Total charge, $, median (IQR)	72,428 (41,994-120,942)	85,318 (53,840-138,278)	13.7	<0.001	MWU
Length of stay, d, median (IQR)	4 (2-7)	4 (3-8)	12.3	<0.001	MWU
Lowest ZIP income quartile, n (%)	15,207 (27.7)	2,063 (29.2)	7.0	0.008	χ²
Large hospital bed size, n (%)	31,731 (57.8)	4,197 (59.4)	6.6	0.010	χ²
Urban teaching hospital, n (%)	44,961 (81.9)	5,956 (84.3)	24.7	<0.001	χ²
Elective admission, n (%)	22,508 (41)	3,744 (53)	368.8	<0.001	χ²
In-hospital death, n (%)	3,746 (6.8)	278 (3.9)	86.0	<0.001	χ²

Comorbidity profile

The obese cohort carried a heavier metabolic and cardiovascular comorbidity signature (Table 2). Relative to non-obese patients, obese patients had higher prevalences of hypertension (3,681 (52.1%) vs. 25,143 (45.8%)), diabetes (1,286 (18.2%) vs. 5,874 (10.7%)), and dyslipidemia (4,239 (60%) vs. 25,418 (46.3%)), together with a 4.5-fold higher prevalence of obstructive sleep apnea (1,703 (24.1%) vs. 2,910 (5.3%)). Cardiovascular conditions were similarly more common, including congestive heart failure (1,335 (18.9%) vs. 7,137 (13%)), atrial fibrillation (1,547 (21.9%) vs. 10,211 (18.6%)), and prior percutaneous coronary intervention (537 (7.6%) vs. 3,513 (6.4%)). Tobacco use coding was essentially identical (5,051 (71.5%) vs. 39,142 (71.3%); p=0.740), and deficiency anemia (283 (4%) vs. 2,745 (5%)) and alcohol abuse (170 (2.4%) vs. 2,196 (4%)) were modestly less frequent in the obese group.

**Table 2:** Selected comorbidities and in-hospital complications Data are presented as n (%) unless stated. Categorical variables were compared using the Pearson chi-squared (χ²) test; the weighted Elixhauser score (continuous) was compared using the Mann-Whitney U test. Note the divergence between comorbidity count (higher in obese) and weighted Elixhauser score (lower in obese).

Variable	Non-obese (N=54,898), n (%)	Obese (N=7,065), n (%)	Test statistic	P-value
Hypertension, uncomplicated	25,143 (45.8)	3,681 (52.1)	99.9	<0.001
Diabetes, uncomplicated	5,874 (10.7)	1,286 (18.2)	344.7	<0.001
Dyslipidemia	25,418 (46.3)	4,239 (60)	470.8	<0.001
Obstructive sleep apnea	2,910 (5.3)	1,703 (24.1)	3212.1	<0.001
Congestive heart failure	7,137 (13)	1,335 (18.9)	184.3	<0.001
Cardiac arrhythmia	14,493 (26.4)	2,014 (28.5)	14.2	<0.001
Atrial fibrillation	10,211 (18.6)	1,547 (21.9)	44.2	<0.001
Known coronary artery disease	11,419 (20.8)	1,809 (25.6)	86.1	<0.001
Prior percutaneous coronary intervention	3,513 (6.4)	537 (7.6)	14.8	<0.001
Pulmonary hypertension	1,867 (3.4)	311 (4.4)	18.5	<0.001
Tobacco use	39,142 (71.3)	5,051 (71.5)	0.1	0.740
Deficiency anemia	2,745 (5)	283 (4)	13.3	<0.001
Alcohol abuse	2,196 (4)	170 (2.4)	43.3	<0.001
Renal failure	6,588 (12)	1,180 (16.7)	126.2	<0.001
Hepatic failure	2,580 (4.7)	417 (5.9)	19.7	<0.001
Fluid/electrolyte disorder	17,952 (32.7)	1,999 (28.3)	55.7	<0.001
Major bleeding	4,721 (8.6)	551 (7.8)	5.2	0.019
Acute myocardial infarction	1,043 (1.9)	92 (1.3)	12.4	<0.001
Respiratory failure	5,325 (9.7)	714 (10.1)	1.2	0.256
Weighted Elixhauser score, median (IQR)	16 (7-24)	10 (3-19)	-28.5	<0.001

An important nuance concerns aggregate comorbidity burden. Obese patients had a greater number of Elixhauser comorbidities, yet a lower weighted Elixhauser (van Walraven) score (median: 10 (IQR: 3-19) vs. 16 (IQR: 7-24); p<0.001). This apparent contradiction is not an error: the van Walraven system assigns large positive mortality weights to conditions such as weight loss, fluid/electrolyte disorders, and metastatic disease and a negative weight to obesity itself [22,23]. The lower weighted score in the obese arm therefore reflects a relative under-representation of the high-lethality, wasting-associated diagnoses that dominate the non-obese arm, an observation that is central to interpreting the mortality finding, as addressed in the Discussion.

Multivariable analysis

After adjustment for demographics, socioeconomic status, hospital characteristics, and comorbidities, an obesity code was independently associated with 33% lower odds of in-hospital mortality (aOR: 0.67; 95% CI: 0.58-0.77; p<0.001; Table 3). Other factors associated with lower mortality included female sex (aOR: 0.83; 95% CI: 0.77-0.89), dyslipidemia (aOR: 0.73; 95% CI: 0.67-0.79), and elective admission (aOR: 0.26; 95% CI: 0.23-0.29). Conversely, the strongest predictors of death were major adverse cardiac events (aOR: 48.88; 95% CI: 38.63-62.39), acute myocardial infarction (aOR: 2.68; 95% CI: 2.04-3.50), fluid/electrolyte disorders (aOR: 2.16; 95% CI: 2.01-2.32), hepatic failure (aOR: 1.46; 95% CI: 1.27-1.67), and cardiac arrhythmia (aOR: 1.52; 95% CI: 1.35-1.70).

**Table 3:** Multivariable logistic regression for in-hospital mortality (selected covariates) Estimates are from a multivariable logistic regression model fitted to unweighted discharge records; the Wald z statistic is reported with each p-value. Male sex is the reference category for the sex covariate (aOR=1.00). Covariates are grouped by whether they were present on or before admission. Conditions of uncertain timing cannot be reliably separated in the National Inpatient Sample into pre-existing conditions versus events arising during the index hospitalization; estimates for these variables are susceptible to overadjustment and are reported for transparency only, not as baseline predictors of mortality. *Estimates that are physiologically implausible and likely artefactual, reflecting sparse cells, collinearity, or definitional overlap with the outcome; reported for transparency only and not interpreted.

Variable	aOR	95% CI	Wald z	P-value
Baseline characteristics and pre-existing comorbidities				
Male sex (reference)	1.00	-	-	-
Obesity (documented, vs. no obesity code)	0.67	0.58-0.77	-5.5	<0.001
Female sex (vs. male)	0.83	0.77-0.89	-5.0	<0.001
Dyslipidemia	0.73	0.67-0.79	-7.5	<0.001
Elective admission	0.26	0.23-0.29	-22.8	<0.001
Hypertension, uncomplicated	0.82	0.75-0.89	-4.5	<0.001
Prior percutaneous coronary intervention	0.72	0.60-0.86	-3.6	<0.001
Alcohol abuse	0.64	0.53-0.77	-4.7	<0.001
Urban teaching hospital	0.59	0.51-0.67	-7.6	<0.001
Congestive heart failure	1.42	1.06-1.87	2.4	0.016
Cardiac arrhythmia	1.52	1.35-1.70	7.1	<0.001
Hepatic failure	1.46	1.27-1.67	5.4	<0.001
Conditions of uncertain timing (not interpreted as baseline predictors)				
Major bleeding	1.77	1.59-1.97	10.4	<0.001
Fluid/electrolyte disorder	2.16	2.01-2.32	21.0	<0.001
Acute myocardial infarction	2.68	2.04-3.50	7.2	<0.001
Major adverse cardiac events*	48.88	38.63-62.39	31.8	<0.001
Stroke*	0.033	0.018-0.058	-11.4	<0.001

Two coefficients warrant explicit caution. The odds ratio for a stroke diagnosis (aOR: 0.033) and that for major adverse cardiac events (aOR: 48.88) are both far outside a physiologically plausible range and almost certainly reflect small-cell instability, collinearity, or partial definitional overlap between predictor and outcome rather than true effects. These estimates are reported for transparency but are not interpreted.

## Discussion

In this nationwide analysis of nearly 62,000 lung cancer hospitalizations, a coded diagnosis of obesity was independently associated with a one-third reduction in the odds of in-hospital death. On its face, this reproduces the widely reported obesity paradox and aligns with surgical, immunotherapy, and meta-analytic literature describing better lung cancer outcomes at higher BMI [7,8,10-12]. However, attributing this observed survival benefit to a direct protective effect of adiposity may be premature and warrants cautious interpretation. The remainder of this discussion examines the competing explanations.

Biological explanations that are genuinely possible

Several mechanisms could plausibly confer a survival advantage on some obese patients. Greater energy and protein reserves may buffer the catabolic stress of acute illness and cancer treatment; the concept of "metabolically healthy obesity" identifies a subgroup with preserved metabolic and immune homeostasis and lower cancer mortality; adipose-derived signalling may modulate the tumor immune microenvironment and checkpoint inhibitor responsiveness; and higher BMI often co-travels with greater skeletal muscle mass, which is itself prognostic [10,12,24]. These are not fanciful hypotheses, and body composition studies suggest that muscle, not fat, may carry much of the apparent benefit [24]. If real, they would argue for measuring body composition directly rather than relying on BMI.

Why the paradox is more likely an artefact

An alternative and arguably stronger explanation is that the association is generated by the structure of the data. Four issues converge.

First, obesity is severely under-captured in administrative data. Validation studies repeatedly show that ICD-based obesity codes have high specificity but very low sensitivity, frequently in the range of 3-19%, so that most truly obese patients are never coded as such [25]. In our cohort, only 11.4% were coded obese, against a true US adult prevalence exceeding 40%. The patients who do get coded are a non-random minority, typically those in whom obesity is clinically salient (e.g., in the context of sleep apnea or surgical planning). This differential capture can by itself distort the exposure-outcome relationship in unpredictable directions.

Second, the reference group is fatally heterogeneous. A binary obese/non-obese split lumps genuinely normal-weight patients together with underweight and cachectic patients. In advanced cancer, low body weight is frequently a manifestation of cachexia, an inflammatory, wasting syndrome that is one of the most powerful independent predictors of death, drives treatment intolerance, and precedes mortality by months [26,27]. A "protective" effect of obesity may therefore be nothing more than the lethal effect of cachexia hiding inside the comparison group.

Third, this cohort is exactly the setting in which collider-stratification and selection bias flourish. Conditioning on hospitalization for lung cancer, a shared downstream consequence of both obesity and competing causes of frailty and death, can induce a spurious inverse association between obesity and mortality even when none exists or reverse the direction of a truly harmful effect [16-19]. The obesity paradox is the canonical textbook example of this phenomenon, and a hospitalized, prevalent (rather than incident) cohort maximizes the risk.

Fourth, the internal evidence points the same way. The obese group carried more comorbidities by count yet a lower weighted Elixhauser score, precisely because the weighting scheme loads heavily on wasting- and severity-associated diagnoses that concentrate in the non-obese arm [22,23]. In other words, the leaner patients carried a greater burden of the conditions most strongly associated with in-hospital death. The mortality difference and the weighted comorbidity difference are two faces of the same confounding, not independent confirmation of a protective effect.

What in-hospital mortality can and cannot tell us

Even setting bias aside, the outcome is limited. In-hospital mortality during a single admission is not cancer survival. The National Inpatient Sample contains no information on tumor stage, histology, performance status, smoking pack-years, systemic therapy, or do-not-resuscitate and hospice status, all of which are decisive for lung cancer prognosis and are plausibly imbalanced between groups [15,20]. Total charges, moreover, are not costs and partly reflect coding intensity rather than resource use. With more than 60,000 records, trivial differences, such as the 0.2-point difference in tobacco coding, reach "significance" without clinical meaning, and several model coefficients (a stroke aOR of 0.03 and a MACE aOR of 49) are physiologically implausible and indicate sparse cell instability or definitional overlap rather than a biological effect.

Clinical implications

The responsible reading of these data is cautionary, not reassuring. Nothing here justifies telling an obese patient with lung cancer that their weight is protective nor deprioritizing nutritional, functional, or oncological optimization on the strength of a favorable odds ratio. If anything, the analysis argues that BMI, and certainly a binary obesity code, is the wrong variable. Prognostic models for lung cancer should incorporate direct measures of body composition (e.g., CT-derived skeletal muscle and adipose indices), continuous BMI with an explicit underweight/cachexia category, tumor stage, and performance status [24,26]. Only then can the question of whether adiposity is protective, or low body weight harmful, be reliably answered.

Strengths and limitations

The principal strength is scale: a large, all-payer sample drawn from a nationally representative sampling frame that captures real-world lung cancer admissions across the United States and is well suited to describing inpatient patterns [20,21]. The limitations, however, are substantial and largely intrinsic to the design. Obesity is defined by low-sensitivity coding; the exposure is binary and cannot separate normal-weight from cachectic patients; the design is cross-sectional and prevalent, maximizing collider and reverse-causation bias; the outcome is single-admission mortality rather than survival; key oncological covariates (stage, histology, therapy, and performance status) and end-of-life variables (do not resuscitate and hospice) are unmeasured; and each record is a hospitalization rather than a patient, allowing double-counting. These constraints do not merely add noise; they plausibly generate the paradox itself.

Three analytic limitations warrant particular emphasis. First, the analysis was performed on unweighted discharge records without incorporating the National Inpatient Sample complex survey design. Because discharge weights, sampling strata, and hospital clustering were not applied, the figures reported here characterize the sampled discharges rather than the US inpatient population, and standard errors are likely underestimated because the clustered, stratified sampling structure is unaccounted for. The reported confidence intervals and p-values should therefore be regarded as narrower than design-based estimation would yield, and the point estimates should not be read as national estimates. Replication using survey-weighted estimation is needed before these findings are generalized.

Second, the National Inpatient Sample does not consistently record present-on-admission status for several diagnoses included in the model. Variables such as major adverse cardiac events, stroke, acute myocardial infarction, major bleeding, fluid and electrolyte disorders, and respiratory failure may therefore represent events occurring during the index hospitalization rather than pre-existing comorbidities. Adjusting for such events, which lie on the causal pathway between admission and death, risks overadjustment and can distort the estimated association between obesity coding and mortality. We have grouped these variables separately in Table 3 and refrain from interpreting them, but we cannot exclude the possibility that their inclusion has biased the adjusted obesity estimate to an unknown degree.

Third, the extreme estimates for major adverse cardiac events and stroke are physiologically implausible and indicate model instability, most plausibly arising from sparse cells, collinearity among overlapping cardiovascular definitions, or partial definitional overlap between these predictors and the mortality outcome. Formal model diagnostics, including assessment of multicollinearity, evaluation of sparse cell and separation problems, and sensitivity analyses restricting the model to baseline comorbidities, were not performed for the present analysis. Until such diagnostics and a re-specified model excluding conditions of uncertain timing are available, the adjusted obesity estimate should be regarded as provisional rather than robust, and the association should be interpreted as involving documented obesity coding rather than adiposity itself.

## Conclusions

Among lung cancer hospitalizations in the 2020-2022 National Inpatient Sample, documented obesity coding was independently associated with lower in-hospital mortality. Considered in isolation, this finding is consistent with previous reports of the obesity paradox. Considered critically, it illustrates the recognized sources of that paradox: an exposure that is under-coded and non-randomly captured, a comparison group contaminated by cachexia, an outcome that is not survival, and a prevalent hospitalized cohort that is the archetypal breeding ground for collider bias. The internal data, more comorbidities but a lower weighted severity score in the obese arm, reinforce rather than refute this interpretation, indicating that the leaner patients carried a greater burden of conditions associated with acute mortality. We therefore conclude that these findings represent an association involving documented obesity coding rather than an effect of adiposity itself and should not be interpreted as evidence that obesity protects patients with lung cancer. They should instead motivate studies built to answer the causal question honestly: incident (not prevalent) cohorts, survey-weighted estimation that reflects the sampling design, models restricted to covariates of confirmed baseline timing, continuous body composition metrics rather than a binary code, explicit adjustment for cachexia and cancer stage, and longitudinal survival endpoints. Until such data exist, the obesity paradox in lung cancer is best treated as a hypothesis about measurement and bias, not a fact about biology.
